# Enhanced Efficiency and Mechanical Stability in Flexible Perovskite Solar Cells via Phenethylammonium Iodide Surface Passivation

**DOI:** 10.3390/nano15141078

**Published:** 2025-07-11

**Authors:** Ibtisam S. Almalki, Tamader H. Alenazi, Lina A. Mansouri, Zainab H. Al Mubarak, Zainab T. Al Nahab, Sultan M. Alenzi, Yahya A. Alzahrani, Ghazal S. Yafi, Abdulmajeed Almutairi, Abdurhman Aldukhail, Bader Alharthi, Abdulaziz Aljuwayr, Faisal S. Alghannam, Anas A. Almuqhim, Huda Alkhaldi, Fawziah Alhajri, Nouf K. AL-Saleem, Masfer Alkahtani, Anwar Q. Alanazi, Masaud Almalki

**Affiliations:** 1 Future Energy Technologies Institute, King Abdulaziz City for Science and Technology (KACST), P.O. Box 6086, Riyadh 11442, Saudi Arabia; eb.5555m@gmail.com (I.S.A.); s.alenzi@kacst.gov.sa (S.M.A.); yalzhrani@kacst.gov.sa (Y.A.A.); aaldukhail@kacst.gov.sa (A.A.); balarthi@kacst.gov.sa (B.A.); aaljuweir@kacst.gov.sa (A.A.); fsalghannam@outlook.com (F.S.A.); amukhem@kacst.gov.sa (A.A.A.); mqhtani@kacst.gov.sa (M.A.); 2 Department of Physics, College of Science and Humanities, Imam Abdulrahman Bin Faisal University, P.O. Box 1982, Jubail 35811, Saudi Arabia; 2170000409@iau.edu.sa (T.H.A.); 2210003660@iau.edu.sa (L.A.M.); 2200000504@iau.edu.sa (Z.H.A.M.); 2210003664@iau.edu.sa (Z.T.A.N.); hsalkaldi@iau.edu.sa (H.A.); fsalhajri@iau.edu.sa (F.A.); 3 Department of Chemistry, King Saud University, P.O. Box 2455, Riyadh 11451, Saudi Arabia; ghazalyafi@gmail.com; 4 Quantum Technologies & Advanced Computing Institute, King Abdulaziz City for Science and Technology (KACST), P.O. Box 6086, Riyadh 11442, Saudi Arabia; azalmutairi@kacst.gov.sa

**Keywords:** flexible perovskite solar cells (FPSCs), surface passivation, phenethylammonium iodide (PEAI), mechanical durability, power conversion efficiency (PCE), polyethylene terephthalate (PET)

## Abstract

Flexible perovskite solar cells (FPSCs) hold great promise for lightweight and wearable photovoltaics, but improving their efficiency and durability under mechanical stress remains a key challenge. In this work, we fabricate and characterize flexible planar FPSCs on a polyethylene terephthalate (PET). A phenethylammonium iodide (PEAI) surface passivation layer is introduced on the perovskite to form a two-dimensional capping layer, and its impact on device performance and stability is systematically studied. The champion PEAI-passivated flexible device achieves a power conversion efficiency (PCE) of ~16–17%, compared to ~14% for the control device without PEAI. The improvement is primarily due to an increased open-circuit voltage and fill factor, reflecting effective surface defect passivation and improved charge carrier dynamics. Importantly, mechanical bending tests demonstrate robust flexibility: the PEAI-passivated cells retain ~85–90% of their initial efficiency after 700 bending cycles (radius ~5 mm), significantly higher than the ~70% retention of unpassivated cells. This work showcases that integrating a PEAI surface treatment with optimized electron (SnO_2_) and hole (spiro-OMeTAD) transport layers (ETL and HTL) can simultaneously enhance the efficiency and mechanical durability of FPSCs. These findings pave the way for more reliable and high-performance flexible solar cells for wearable and portable energy applications.

## 1. Introduction

Flexible perovskite solar cells (FPSCs) have emerged as a cutting-edge photovoltaic technology, offering the PCEs of perovskite materials in lightweight, bendable formats [1,2,3,4,5]. The urgency for renewable and portable energy solutions, driven by global energy demand and the limitations of conventional rigid silicon solar panels, has intensified research into flexible photovoltaics. Rigid perovskite solar cells (PSCs) based on metal halide perovskites (ABX_3_ structures) have achieved remarkable efficiency gains in the past decade, now rivaling established PV technologies with laboratory PCEs over 26% [6,7]. Early demonstrations of FPSCs, however, showed modest efficiencies; for example, Kumar et al. (2013) reported a PCE of only ~2.6% for a flexible device [8]. Thanks to advances in materials and interfaces, recent flexible devices have reached much higher efficiencies [9,10,11,12,13]. Notably, some state-of-the-art flexible perovskite cells can maintain most of their initial efficiency even after many bending cycles, highlighting substantial progress in mechanical resilience [14]. These trends underscore the potential of FPSCs to become competitive with traditional solar modules, enabling solar power in applications where rigid panels are impractical (wearables, roll-up devices, aerospace, etc.) [15,16,17,18,19].

Despite their promise, FPSCs face unique challenges related to both device architecture and stability. The choice of flexible substrate is critical: common polymer films like polyethylene terephthalate (PET) and polyethylene naphthalate (PEN) offer transparency, light weight, and flexibility, but must withstand the processing temperatures of device fabrication [20,21,22,23,24,25,26,27]. PET can tolerate temperatures up to ~150 °C and serves as a mechanically robust support for the thin-film stack [28,29,30,31,32]. Another challenge is engineering each layer of the solar cell for flexibility without compromising performance. Transparent electrodes, such as indium tin oxide (ITO), are standard in rigid cells but tend to crack under strain; nevertheless, with careful handling, ITO-coated PET can be employed for lab-scale flexible cells. Charge transport layers must also be optimized for the flexible context. Tin oxide (SnO_2_) has become a popular ETL for high-efficiency PSCs due to its suitable band alignment and low-temperature processability [33,34,35,36,37]. However, differences in surface properties between plastic and rigid substrates demand careful tuning of such ETLs; studies have shown that achieving >15% efficient FPSCs requires ETLs tailored to plastic substrates [38,39]. On the hole transport side, spiro-OMeTAD (doped with lithium bis(trifluoromethanesulfonyl)imide and 4-tert-butylpyridine) remains a benchmark material delivering reliable performance, though its hygroscopic dopants can impact long-term stability [40,41,42].

A particularly important aspect for improving both efficiency and stability is interface engineering of the perovskite active layer. Thin-film halide perovskites are prone to defects at grain boundaries and surfaces, which act as non-radiative recombination centers and initiation points for degradation (e.g., under moisture or bias stress) [43,44,45]. Significant advances in perovskite photovoltaics, including optimized interface engineering and passivation strategies, have driven efficiency improvements. Recent work on coherent interface formation in perovskite solar cells highlights effective strategies to enhance charge extraction and reduce interface defects, directly contributing to higher open-circuit voltages and overall device performance [46]. Additionally, developments in tandem structures incorporating perovskite and organic semiconductors emphasize the importance of versatile interfacial strategies for multi-functional photovoltaic devices [47]. Orientation control and the engineering of thin-film interfaces play critical roles in determining the photovoltaic properties of layered materials. Advanced deposition techniques, such as those demonstrated for oriented Pb-based thin films, have underscored the correlation between crystallographic orientation, interfacial quality, and dielectric properties [48]. Similarly, low-temperature synthesis approaches developed for dielectric oxide films highlight the value of controlled processing conditions in obtaining defect-minimized thin-film materials [49]. Surface passivation strategies using large organic cations have proven effective in addressing these issues [50,51,52]. Phenethylammonium iodide (PEAI) is one such organic salt that has been widely used to form a two-dimensional (2D) perovskite layer at the surface of three-dimensional (3D) perovskite films [53,54,55]. The bulky phenethylammonium (PEA^+^) cation can terminate the perovskite lattice, creating a thin PEA_2_PbI_4_-like 2D capping layer that electronically passivates dangling bonds and defect sites on the surface. This 2D/3D interface has been shown to reduce surface recombination, leading to higher open-circuit voltage and fill factor in devices [56,57,58,59,60]. Additionally, the hydrophobic organic cations can act as a barrier to moisture ingress, improving environmental stability. By healing defects and preventing charge traps, PEAI and similar large-cation post-treatments have enabled notable efficiency improvements and increased operational stability in PSCs. In flexible cells, these benefits could extend further to mechanical stability: a well-passivated, quasi-2D surface may better tolerate strain by preventing crack initiation at grain boundaries. There is growing evidence that such interface layers can enhance mechanical durability; for instance, devices with certain 2D interfaces or additives have retained >90% efficiency after thousands of bending cycles.

The bending stability of flexible perovskite solar cells (FPSCs) is a critical determinant of their practical applicability in portable and wearable electronics. Recent investigations have demonstrated that although well-optimized FPSCs can retain over 90% of their original efficiency after numerous bending cycles, the underlying mechanisms are governed by a complex interplay of mechanical strain, optical modulation, and interfacial delamination [61,62]. For instance, Du et al. showed that when FPSCs are bent to a radius of 90°, they maintain up to 95.12% of their initial power conversion efficiency (PCE), and the bending direction significantly influences device performance with upward bending producing up to 9.9% higher current density than downward bending due to light convergence and enhanced carrier generation near the bending center [63]. Finite element modeling further reveals that stress tends to localize in stiff layers like ITO and Ag, often leading to cracking and electrical failure upon cyclic loading. To mitigate these effects, flexible transparent electrodes, such as PH1000, and engineering approaches, like neutral axis repositioning using protective layers, have been explored. Devices incorporating PH1000 exhibited minimal resistance variation (<14%) and retained over 60% of PCE after 2000 cycles at a tight 2 mm bending radius, in contrast to the catastrophic failure observed in ITO-based counterparts [64]. Such findings emphasize the need for an integrative design approach encompassing material selection, structural reinforcement, and stress-distribution tuning to ensure durable mechanical performance under realistic flexing conditions. While previous studies have extensively addressed PEAI passivation in rigid perovskite systems, detailed investigations into its mechanical and photovoltaic implications on flexible substrates remain limited. Addressing this gap, the current work systematically examines PEAI passivation’s impact on mechanical robustness and photovoltaic performance of flexible perovskite solar cells, thereby contributing practical insights and extending previous interfacial engineering concepts into the flexible device regime [46,47].

In this study, we focus on a flexible perovskite solar cell with a double-cation mixed-halide perovskite absorber (composition (MACl)_0.33_(FAPbI_3_)_0.97_(MAPbBr_3_)_0.1_) and investigate the effects of a PEAI surface treatment on its performance and bending stability. To test this, we fabricate devices on PET/ITO substrates with and without the PEAI treatment and carry out comprehensive characterization: structural (XRD, SEM), optical (UV–Vis absorption and photoluminescence, PL), photovoltaic (*J*–*V* characteristics under simulated sunlight), and mechanical (bending cycle tests). The results demonstrate that PEAI passivation increases the PCE of our flexible cells by mitigating interfacial recombination, and more strikingly, it significantly enhances mechanical durability under cyclic strain. By integrating literature insights and our experimental findings, we discuss how this surface engineering approach can address key bottlenecks in FPSC performance. Our work contributes to the ongoing efforts to develop flexible perovskite photovoltaics that are not only efficient but also robust enough for real-world applications, moving FPSCs closer to commercialization.

## 2. Methodology

### 2.1. Device Fabrication

**Figure 1:**
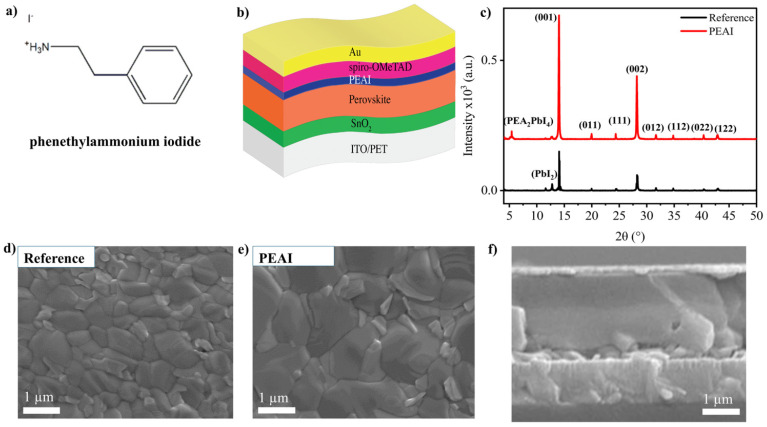
(**a**) Molecular structure of polyethylene terephthalate (PET) and phenethylammonium iodide (PEAI). (**b**) A schematic representation of the FPSC. (**c**) XRD diffraction pattern of reference and PEAI-treated perovskite film. Top-view SEM images of (**d**) reference and (**e**) PEAI-treated perovskite films. (**f**) Cross-sectional SEM image of PEAI-treaed perovskite film.

Flexible substrates of PET (Figure 1a) coated with indium tin oxide (ITO) were used as the transparent bottom electrode. The PET/ITO substrates (dimensions ~2.5 cm × 2.5 cm, sheet resistance ~100 Ω/□) were cleaned sequentially with detergent, deionized water, ethanol, and isopropanol, and then dried with nitrogen. A UV-ozone treatment for 15 min was applied to improve surface cleanliness and wettability prior to layer deposition. A compact SnO_2_ ETL was deposited on the ITO-coated PET via spin coating. We employed a commercially available SnO_2_ colloidal precursor (2.5% in water) diluted as necessary. The SnO_2_ solution was dispensed onto the substrate and spun at 4000 rpm (acceleration 2000 rpm/s) for 20 s, yielding a thin, uniform film. The films were then annealed at 150 °C for 60 min on a hotplate to densify the SnO_2_ layer and enhance its conductivity. This low-temperature process is compatible with PET and results in a transparent n-type layer that facilitates electron extraction from the perovskite. The thickness of the SnO_2_ ETL is on the order of ~30–50 nm (as observed in cross-section SEM). A double-cation lead mixed-halide perovskite with nominal formula (MACl)_0.33_(FAPbI_3_)_0.97_(MAPbBr_3_)_0.1_ was prepared via a one-step spin-coating process in a nitrogen glovebox. The precursor solution was formulated by dissolving the appropriate stoichiometric amounts of methylammonium chloride, methylammonium bromide (MABr), formamidinium iodide (FAI), lead iodide (PbI_2_), and lead bromide (PbBr_2_) in a mixed solvent of N,N-dimethylformamide (DMF) and dimethylsulfoxide (DMSO). Specifically, we used a recipe of: 33 mg MACl, 247 mg FAI, 5 mg MABr, 722.4 mg PbI_2_, and 16.5 mg PbBr_2_, dissolved in 1 mL of DMF:DMSO (4:1 *v*/*v*). The solution was spin-coated in two steps: 2000 rpm for 10 s, followed by 6000 rpm for 30 s. During the second step, an anti-solvent (chlorobenzene, ~200 µL) was dripped onto the spinning substrate ~12 s before the end of the spin cycle. The anti-solvent induces rapid crystallization of the perovskite, resulting in uniform film formation. Immediately after spin coating, the films were annealed at 120 °C for 25 min. For selected samples, a surface passivation layer was applied to the perovskite film after annealing. A PEAI (Figure 1a) solution (6 mg mL^−1^ in isopropanol) (approximately 0.02 M) was prepared and dynamically spin-coated onto the cooled perovskite film at 3000–4000 rpm for 10–20 s. This step yields two sets of samples: “w/o PEAI” (no passivation) and “with PEAI” (passivated). Next, the hole transport layer was deposited. Spiro-OMeTAD [2,2′,7,7′-tetrakis(N,N-di-p-methoxyphenylamine)-9,9′-spirobifluorene] was used as the HTL, prepared with standard dopants to ensure adequate conductivity. In a nitrogen glovebox, spiro-OMeTAD powder (≈73 mg mL^−1^) was dissolved in chlorobenzene with additives: lithium bis(trifluoromethanesulfonyl)imide (Li-TFSI) solution (520 mg mL^−1^ in acetonitrile, 21.36 µL added) and 4-tert-butylpyridine (tBP, 34.5 µL added). The resulting doped spiro-OMeTAD solution (~70 mM spiro) was spin-coated onto the perovskite (or PEAI-treated perovskite) at 4000 rpm for 20 s. This produces a ~150–200 nm thick transparent HTL. Finally, a gold back contact was deposited to complete the device. Gold (~90 nm thick) was thermally evaporated under high vacuum (<10^−5^ Torr) onto the spiro-OMeTAD layer through a shadow mask. A schematic representation of the full device is illustrated in Figure 1b.

### 2.2. Characterization Techniques

X-ray diffraction (XRD): The crystalline structure of the perovskite films (with and without PEAI) was analyzed by XRD using a Bragg–Brentano θ-2θ geometry (Cu Kα radiation, λ = 1.5406 Å). Scans were recorded from 10° to 40° 2θ at a step of 0.02° to identify the prominent crystallographic peaks of the perovskite.

X-ray photoelectron spectroscopy (XPS) was performed using monochromatic Al Kα radiation (1486.6 eV) to investigate the chemical interactions at the perovskite/PEAI interface, particularly assessing changes in binding energies and chemical environments associated with surface passivation. Spectra were calibrated using the adventitious carbon peak (C 1s) at 284.8 eV as the internal reference. High-resolution scans were collected for Pb 4f, I 3d, and N 1s core levels to elucidate the chemical state modifications resulting from the PEAI treatment.

Ultraviolet Photoelectron Spectroscopy (UPS) measurements were conducted to investigate the energy-level alignment and work function changes at the perovskite/charge transport interfaces. A He I photon source (21.22 eV) was used, and the spectra were acquired under ultra-high vacuum conditions (~10^−8^ Torr). The work function and valence band maximum (VBM) positions were determined from the secondary electron cutoff and valence band edge onset, respectively.

Scanning electron microscopy (SEM): The morphology of the perovskite layers was observed by SEM. Top-view SEM images were taken to evaluate grain size, surface coverage, and the presence of pinholes or defects. Cross-sectional SEM images were also obtained by cleaving devices and imaging the layer stack at a tilted angle.

Optical measurements: UV–Vis absorbance spectra were recorded for the perovskite films (on glass or PET/ITO substrates) using a UV–Vis spectrophotometer in the 400–800 nm wavelength range. The absorption onset (band edge) provides an estimate of the perovskite bandgap. Photoluminescence (PL) spectra were measured using a fluorescence spectrometer (or a custom setup with a 532 nm excitation laser and CCD detector) in ambient conditions. Steady-state PL was collected from films with and without PEAI to gauge differences in radiative recombination.

Current–voltage (*J*–*V*) characteristics: The photovoltaic performance of completed devices was measured under simulated AM 1.5G sunlight. A solar simulator with a 100 mW cm^−2^ illumination (calibrated with a silicon reference cell) was used. The *J*–*V* curves were recorded using a source meter (Keithley 2400) by sweeping the voltage from 0 V to ~1.2 V with a scan rate of ~100 mV/s^−1^ with a delay time of 10 ms at each point. The cells were masked to a defined active area (~0.16 cm^2^) to avoid edge effects and ensure accurate current density calculations. We report the champion and average PCE values. All measurements were performed in ambient air at ~25 °C and relative humidity < 30% to minimize moisture-related degradation during testing.

Mechanical bending test: To evaluate mechanical stability, a cyclic bending test was carried out on the flexible devices. Each device was repeatedly bent inward (concave facing the perovskite side) to a bending radius of approximately 5 mm (unless otherwise noted) and released, constituting one cycle, and then the device was carefully placed back in the solar simulator and its *J*–*V* curve measured under 1-sun illumination.

## 3. Results and Discussion

### 3.1. Structural Properties (XRD, XPS, and SEM)

XRD analysis confirmed that the films crystalized into the expected perovskite structure with high phase purity (Figure 1c). Both PEAI-passivated and control perovskite films showed dominant diffraction peaks at 2θ ≈ 14.0° and 28.4°, corresponding to the (110) and (220) lattice planes of the tetragonal perovskite crystal (in pseudocubic indexing, these relate to the (100) and (200) reflections) [65]. Additional peaks at ~23.5°, 31.8°, and 40.5° were observed, assignable to higher-order perovskite reflections ((211), (310), and (224) planes, respectively), indicating good crystallinity. Crucially, the XRD patterns did not exhibit a significant peak at 12.6° that would indicate the presence of hexagonal PbI_2_ impurity. In the control (unpassivated) film, a very weak shoulder at approximately ~12.6° could be discerned, suggesting trace amounts of residual PbI_2_ or slight decomposition at grain boundaries. In contrast, the PEAI-treated film’s XRD showed this feature to be suppressed below the noise level, implying that the PEAI treatment helped eliminate residual PbI_2_ by reacting with under-coordinated lead at the surface to form PEA_2_PbI_4_ [66]. The XRD pattern of the PEAI-treated film exhibits low-angle reflections characteristic of PEA_2_PbI_4_, confirming the formation of an n = 1 Ruddlesden–Popper (RP) phase at the perovskite surface (Figure S1). The overall intensity of the perovskite peaks was slightly higher (by ~10%) for the PEAI-passivated sample, and the FWHM of the main (110) peak was marginally narrower. Overall, the PEAI layer significantly enhanced the crystallographic orientation, as evidenced by an intensity ratio increase of approximately 3.17 for the dominant (110) peak, accompanied by a minor peak shift of about −0.02° in 2θ, suggesting slight structural adjustments likely due to surface interactions or lattice strain induced by PEAI (Table S1). These differences suggest that the surface passivation may also lead to improved crystallinity or larger effective grain size, possibly because the PEAI aids in healing surface imperfections and promotes better ordering at the film surface. Complementary XPS analysis further supported the mechanism of PEAI-induced passivation. The Pb 4f XPS spectra revealed a pronounced reduction in the intensity of metallic lead (Pb^0^) signals after PEAI treatment, highlighting the effective passivation of Pb-related defect states. This interaction significantly decreases the density of non-radiative recombination centers, thus contributing to the observed improvements in device performance and crystallinity enhancement.

Top-view SEM images provide complementary information on the perovskite film surface. The control perovskite films exhibited grain sizes on the order of 200–500 nm with full surface coverage (no pinholes), consistent with high-quality solution processing (Figure 1d). The grain boundaries are moderately visible as thin lines, and some small PbI_2_ crystals could be occasionally observed at triple junctions of grain boundaries in the control film. In the PEAI-passivated film, the surface looked smoother and grain boundaries were somewhat less pronounced (Figure 1e). This could be because the PEAI post-treatment forms a thin overlayer that covers the grain boundaries. Indeed, the literature reports have noted that organic cation treatments like PEAI can lead to slightly enlarged apparent grain size and a smoother topography due to the formation of a 2D perovskite at the surface that “fills in” surface gaps [67]. In our case, the difference in grain structure was in favor of the PEAI-treated surface, which appeared to have a larger grain size that was free of any small crystallites or residues and had a uniform contrast in SEM, implying a homogeneous capping layer. The grain size distribution analysis demonstrates a clear increase in the average grain size from approximately 0.6 µm in the reference film to approximately 1.2 µm following PEAI treatment (Figure S2). This enlargement and narrower distribution suggest that PEAI effectively promotes grain growth, leading to enhanced morphological uniformity and potentially improved photovoltaic performance. These morphological observations support the XRD findings that PEAI passivation produces a cleaner, possibly more ordered perovskite surface. Such a surface is expected to have fewer defect sites, which correlates with the improved electronic and optical properties discussed below. Cross-sectional SEM image of a flexible perovskite solar cell (with PEAI passivation), showing the layer structure on the PET/ITO substrate (Figure 1f). The bright top layer is the evaporated Au contact, beneath which the darker spiro-OMeTAD (HTL) and perovskite layers are visible. The SnO_2_ ETL is too thin to clearly distinguish at this scale, but it resides between the perovskite and the bottom ITO electrode (on PET). The total thickness of the device stack (excluding PET) is on the order of 800–900 nm, with the perovskite layer being ~500 nm thick, as indicated by the scale bar. The layers appear conformal and continuous, with no large voids or delamination gaps observed. Notably, the perovskite layer in the PEAI-treated device exhibits a dense and uniform morphology, which is favorable for charge transport.

### 3.2. Optical Properties (UV–Vis and PL)

**Figure 2:**
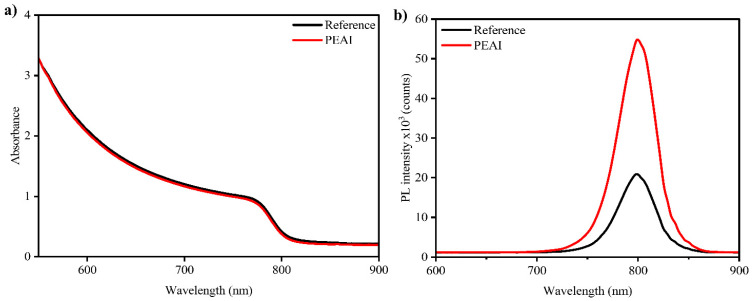
(**a**) UV-Vis and (**b**) PL spectra for the reference and PEAI-passivated perovskite films.

The optical absorption spectra of the perovskite films on glass (both with and without PEAI) are nearly identical in the visible range, indicating that the bulk perovskite remains unchanged by the surface treatment (Figure 2a). The films show a strong absorption onset at approximately 820 nm, corresponding to a bandgap of approximately 1.51–1.55 eV. In the wavelength range of 400–750 nm, the absorbance exceeds 80%, demonstrating the perovskite’s excellent light-harvesting capability over the visible spectrum. Both types of films exhibit a sharp band edge, indicating low Urbach energy (disorder) and high material quality [68]. Photoluminescence measurements, however, reveal a clear difference between passivated and non-passivated films (Figure 2b). Under identical excitation conditions, the PEAI-treated perovskite film exhibits a significantly stronger PL emission at ~800 nm (near the band-edge peak) compared to the untreated film. The integrated PL intensity of the PEAI-passivated sample is roughly double that of the control. This enhancement in PL indicates a lower density of non-radiative recombination centers in the presence of the PEAI layer. Surface defects, which act as traps for charge carriers and quench PL, have been effectively passivated by the phenethylammonium cations. The optical characterization provides evidence that the PEAI treatment passivates surface defects, as seen by the boosted PL intensity. This is a strong indication that solar cells made from these films should exhibit higher open-circuit voltage and reduced recombination losses, since non-radiative recombination at surfaces is a major factor limiting *V_OC_* in perovskite solar cells [55,69,70].

### 3.3. Photovoltaic Performance (J–V Characteristics)

**Figure 3:**
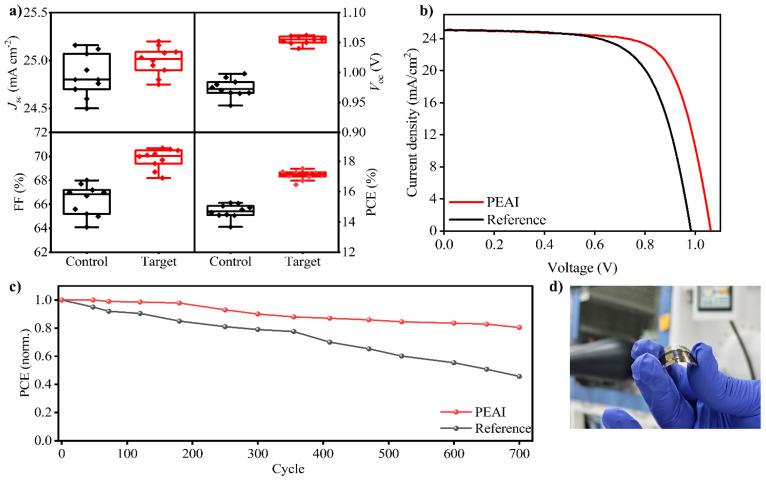
(**a**) Statistical representation of photovoltaic parameters, (**b**) J-V curve of the champion devices. (**c**) Stress bending test of the reference and PEAI-passivated FPSCs. (**d**) A photograph of a bent PEA-passivated FPSC.

The photovoltaic performance of flexible perovskite devices was measured under standard test conditions (1 sun, AM 1.5G). Without the PEAI passivation, the control FPSCs yielded an average PCE of approximately 13–14%, with the best cell reaching 14.5% (Figure 3a). These control devices typically showed a *V_OC_* of ~1.00 V, a *J_sc_* of ~24.8 mA cm^−2^, and an FF of ~0.60–0.65. The somewhat modest *V_OC_* (∼1.0 V) and FF (60–65%) in the control devices indicate there is room for reducing recombination and resistive losses, precisely the issues that surface passivation aims to address. Introducing the PEAI surface treatment led to a notable improvement in device metrics. The PEAI-passivated FPSCs achieved PCEs in the range of 15–17%, with champion devices at ~16.5% PCE under forward-scan illumination. Figure 3b shows representative *J*–*V* curves comparing a control device and a PEAI-passivated device. The most pronounced change is an increase in *V_OC_* for the PEAI device: *V_OC_* improved to ~1.08 V (an increase of ~80 mV relative to control). This directly reflects the reduced recombination at the perovskite/HTL interface due to defect passivation, allowing the device to sustain a higher photovoltage. The fill factor also improved, reaching ~70% in passivated cells. Meanwhile, the *J_sc_* of the devices remained high and comparable between passivated and control cells. This indicates that the PEAI layer is not impeding charge collection or optical absorption; carriers can still efficiently reach the contact electrodes. External quantum efficiency (EQE) spectra and integrated current density of reference and PEAI-passivated devices. The PEAI-treated device shows a higher EQE across most of the visible range, with an integrated Jsc of ~25 mA cm^−2^, consistent with the *J*–*V* measurements (Figure S3). Overall, the average PCE gain from PEAI treatment was on the order of 2–3 percentage points absolute. This relative improvement (~20%) aligns well with previous reports of organic halide salt passivation in PSCs [44,71]. The improved performance of the PEAI-passivated flexible perovskite solar cells (FPSCs) can be attributed in part to enhanced radiative recombination characteristics, as evidenced by the time-resolved photoluminescence (TRPL) and photoluminescence quantum yield (PLQY) results. As shown in Figure S4, the PEAI-treated films exhibit a notably shorter carrier lifetime (τ = 12.5 ns) compared to the reference (τ = 28 ns), indicating accelerated radiative recombination due to defect passivation. Additionally, the PLQY increases from 4% to 15%, confirming that a greater fraction of photoexcited carriers recombine radiatively rather than through non-radiative pathways. These findings suggest that PEAI effectively suppresses surface traps and enhances interfacial quality, which translates to higher open-circuit voltage and improved device efficiency in the flexible architecture. While our TRPL and PLQY measurements clearly demonstrate reduced non-radiative recombination through PEAI passivation, further detailed studies assessing recombination dynamics under mechanical stress (e.g., post-bending conditions) are warranted. These investigations would enhance understanding of the mechanistic relationship between mechanical stability and electronic properties in flexible perovskite solar cells. Furthermore, the PEAI treatment significantly affects the energy level alignment at the perovskite interface, as evidenced by Ultraviolet Photoelectron Spectroscopy (UPS) analysis (Figure S5). Specifically, the introduction of PEAI shifted the perovskite film’s Fermi level (E_F_) deeper from 4.51 eV (reference film) to 4.70 eV (PEAI-passivated film). Correspondingly, the valence band maximum (VBM) moved closer to E_F_, decreasing the offset from 1.01 eV to 0.72 eV. These changes result in improved alignment with the HOMO level of Spiro-OMeTAD (HTL), facilitating more efficient hole extraction and reducing energy loss at the perovskite/HTL interface. This improved energy alignment helps explain the observed enhancements in photovoltaic device performance, particularly the increased *V_oc_* and FF.

### 3.4. Mechanical Flexibility and Stability

A primary advantage of FPSCs is their ability to endure bending, enabling deployment in wearable and portable formats. We evaluated the mechanical robustness of our devices by subjecting them to repeated bending and measuring the retention of photovoltaic performance. The initial (unbent) efficiencies have been described above (≈16.5% with PEAI, ≈14% without). After bending the devices (radius ~5 mm) for 700 cycles, the cells were tested again under the same illumination conditions. Strikingly, the PEAI-passivated devices retained approximately 88% of their original PCE on average after 700 cycles. In contrast, the control devices (no PEAI) retained only about 70% of their initial PCE after the same bending treatment. This difference highlights a substantial improvement in mechanical durability imparted by the PEAI surface layer. Specifically, a typical PEAI-treated device degraded from 16.0% initial PCE down to ~14.0% after 700 bends, whereas a control device might go from 14.0% to ~9.8% in the same test. The dominant loss in both cases was a reduction in the fill factor and a slight drop in *V_oc_*, while *J_sc_* remained fairly stable (within 5% of the initial). While 700 cycles are a considerable stress test (far exceeding the bending a typical wearable device might undergo daily), we note that literature reports have demonstrated FPSCs enduring >1000 or even >10,000 bending cycles with specialized architectures. For instance, Li et al. reported flexible cells retaining over 90% of initial efficiency after 1000 bends, and other groups using reinforcing structures have achieved stable performance over 10,000 cycles [72]. Our results, reaching ~88% after 700 cycles without any encapsulation or special structural enhancements, are in line with these findings and confirm that surface passivation is a viable strategy to improve mechanical resilience. Figure 3d shows a photograph of a flexible perovskite solar cell during a bending test (device curvature radius ~5 mm). The cell is bent along the PET substrate, illustrating its mechanical flexibility. Even under significant bending, the layered structure (PET/ITO/SnO_2_/perovskite/spiro/Au) remains intact without visible cracking. The incorporation of the PEAI passivation layer contributes to this robustness by forming a flexible 2D interface that can accommodate strain. The PEAI at the surface likely acts to “bridge” grain boundaries and adhere the perovskite crystals together, reducing the likelihood of mechanical fracture under flexing [73]. Additionally, PEA^+^ cations might impart some ductility at the interface, as 2D perovskites are known to be more pliable compared to 3D perovskites [74]. The outcome is an FPSC that not only performs well initially but also shows enhanced longevity under repetitive bending, a critical attribute for any practical deployment of solar cells on flexible platforms. Figure 3 underlines the potential of such devices for wearable technology, for example, solar textiles or device-integrated solar chargers, where constant movement and bending are expected. The visual integrity of the cell in the bent state correlates with the electrical data, indicating sustained performance. The thermal stability of devices was rigorously examined to assess the efficacy of the PEAI passivation layer under prolonged heating conditions (60 °C) at a relative humidity of ~ 25%. Over a period of 500 h, PEAI-treated devices demonstrated significantly enhanced stability, maintaining roughly 85% of their initial photovoltaic performance, as illustrated in Figure S6. In contrast, untreated control devices exhibited pronounced efficiency loss, retaining only approximately 60% of their original efficiency under identical conditions. This marked difference underscores the effectiveness of PEAI in stabilizing the perovskite interfaces against thermal degradation. Variations across five individual devices per condition were consistently minor, as indicated by the small standard deviations, highlighting the reproducibility and robustness of the PEAI passivation strategy. Overall, the developed flexible perovskite solar cells demonstrated promising mechanical stability, enduring extensive bending tests exceeding 700 cycles at a bending radius of 5 mm without significant performance degradation. This performance surpasses or is comparable to many recent state-of-the-art flexible perovskite devices, highlighting the effectiveness of the implemented PEAI passivation strategy in enhancing both structural robustness and reliability for flexible device applications (Table S2). Compared to recently reported passivation methods such as PMMA, PEABr, BAI, and OAI, our PEAI-treated flexible PSCs demonstrate superior photovoltaic efficiency (17%) and notable mechanical resilience, retaining approximately 88% of their initial efficiency after 700 bending cycles (Table S3). The impressive mechanical robustness demonstrated by PEAI-treated perovskite solar cells under bending conditions is promising for flexible photovoltaic applications. The enhancement in mechanical stability likely originates from the microstructural improvements facilitated by PEAI treatment, as evidenced by larger grain sizes and reduced grain boundary defects, contributing to minimized stress concentration points and crack initiation sites [75,76]. Furthermore, the Ruddlesden–Popper phase generated via PEAI passivation provides additional resistance to moisture ingress and environmental degradation, thus potentially extending device lifetime under operational stresses [77]. Integration of these mechanically robust and microstructurally optimized films into practical device architectures requires attention to interface engineering and encapsulation strategies, where optimal alignment of energy levels and minimized interfacial recombination are critical to achieving high device performance and durability under realistic environmental conditions [78,79].

## 4. Conclusions

We have developed and investigated a flexible perovskite solar cell based on a PET/ITO substrate incorporating a PEAI surface passivation layer. The inclusion of the PEAI passivation layer significantly improves the photovoltaic performance of the flexible cells. PEAI-treated devices achieved PCEs at approximately 16–17%, compared to ~14% for identical devices without PEAI. The improvement is attributed to the effective passivation of surface defects by PEAI, which suppresses nonradiative recombination and yields a more ideal diode behavior. Our results demonstrate that even on flexible substrates, where interfaces may be more defect-prone, applying a 2D cation passivation strategy can push device efficiencies closer to those of rigid, state-of-the-art PSCs. A key outcome of this work is the demonstrated improvement in mechanical stability for PEAI-passivated FPSCs. In bending tests, devices with PEAI retained nearly 90% of their initial efficiency after 700 bending cycles, significantly outperforming unpassivated control devices, which fell to ~70% of their initial efficiency. Improved efficiency and bending durability via a simple surface treatment, which fits well into the larger efforts of making perovskite solar cells more viable in unconventional scenarios. Our findings concur with the growing consensus in the perovskite research community that interface engineering (through chemical means like self-assembled monolayers or 2D cations) is crucial for both achieving high performance and mitigating degradation mechanisms. By demonstrating this on flexible cells, we bridge the gap between materials chemistry and mechanical engineering. The results encourage further interdisciplinary work to optimize the composition of both the perovskite absorber and the flexible substrates/encapsulants to ultimately produce a flexible solar cell that rivals rigid ones in efficiency and exceeds them in functionality.

## Data Availability

The original contributions presented in this study are included in the article/Supplementary Material. Further inquiries can be directed to the corresponding authors.

## Supplementary Materials

The following supporting information can be downloaded at: https://www.mdpi.com/article/10.3390/nano15141078/s1.
