# Supplementary material for: Enhanced Efficiency and Mechanical Stability in Flexible Perovskite Solar Cells via Phenethylammonium Iodide Surface Passivation
Source: Nanomaterials (Basel). 2025 Jul 11;15(14):1078. doi: 10.3390/nano15141078 (PMC12299172; doi:10.3390/nano15141078)
Supplement: Supplementary file 1 [file nanomaterials-15-01078-s001.zip › nanomaterials-3685516-supplementary.pdf]

Enhanced Efficiency and Mechanical Stability in Flexible Perovskite Solar Cells via Phenethylammonium Iodide Surface Passivation

Ibtisam S Almalki[1], Tamader H. Alenazi[2], Lina A. Mansouri[2], Zainab H. Al Mubarak[2], Zainab T. Al Nahab[2], Sultan M. Alenzi[1], Yahya A. Alzahrani[1], Ghazal S Yafi[3], Abdulmajeed Almutairi[4], Abdurhman Aldukhail[1],Bader Alharthi[1], Abdulaziz Aljuwayr[1], Faisal S. Alghannam[1], Anas A. Almuqhim[1], Huda Alkhaldi[2], Fawziah Alhajri[2], , Nouf K. AL-Saleem[2],* Masfer Alkahtani[1], Anwar Q. Alanazi[1],* and Masaud Almalki[1]*

1 Future Energy Technologies Institute, King Abdulaziz City for Science and Technology (KACST), P.O. Box 6086, Riyadh 11442, Saudi Arabia.

2 Department of Physics, College of Science and Humanities, Imam Abdulrahman Bin Faisal University, P.O. Box 1982, Jubail, Saudi Arabia.

3 Department of Chemistry, King Saud University, P.O. Box 2455, Riyadh, 11451, Saudi Arabia.

4 Quantum Technologies & Advanced Computing Institute, King Abdulaziz City for Science and Technology (KACST), P.O. Box 6086, Riyadh 11442, Saudi Arabia.

* *Correspondence to: mhalmalki@kacst.gov.sa, aqalanazi@kacst.gov.sa, and nkalsaleem@iau.edu.sa

Supporting Information

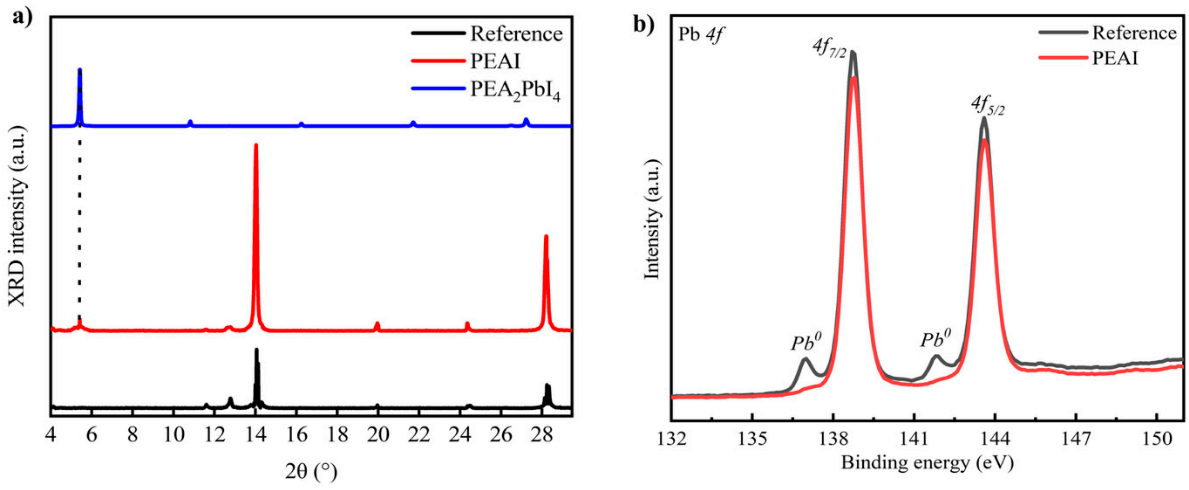

Figure S1. (a) XRD patterns of the reference, PEAI-passivated, and PEA_2PbI_4 films, with the passivated film shown at an angle of 5.5 °, in agreement with PEA_2PbI_4 films. (b) XPS spectra of Pb 4f core levels comparing the reference and PEAI-treated perovskite films. The reduction in the Pb^0 peaks upon PEAI treatment indicates effective chemical passivation of under-coordinated Pb sites, thus confirming suppression of defect states and non-radiative recombination pathways.

Table S1: XRD peak analysis comparing prominent perovskite and PbI_2 diffraction peaks in reference and PEAI-treated films, including peak positions (2θ), intensity ratios, and full-width at half maximum (FWHM), illustrating changes in crystallographic orientation and crystallinity induced by the PEAI layer.

**Peak (Plane/Material)**	**2θ (°)**	**Intensity . 10^3 (Ref.)**	**Intensity . 10^3 (PEAI)**	**Intensity Ratio (PEAI/Ref.)**	**FWHM (Ref.)**	**FWHM (PEAI)**
Perovskite (110)	14.12	720	2283	3.17	0.109	0.089
Perovskite (220)	28.45	398	1095	2.75	0.133	0.117
Perovskite (310)	31.90	285	623	2.19	0.155	0.132
PbI_2	12.68	536	197	0.37	0.224	0.298

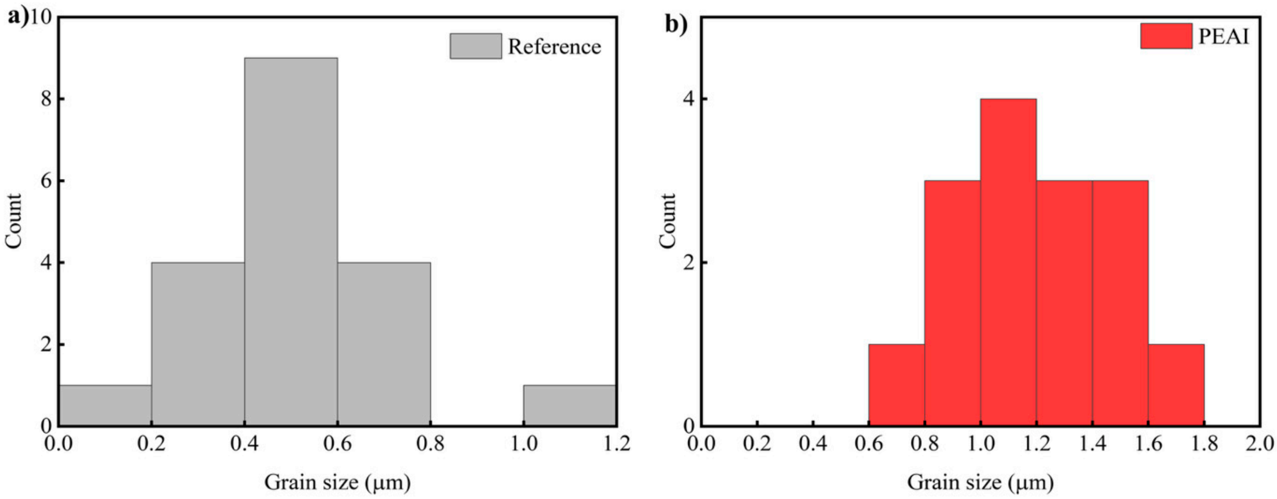

Figure S2. Grain size distribution histograms for (a) reference and (b) PEAI-treated perovskite films, indicating enhanced grain growth and improved morphological uniformity upon PEAI treatment.

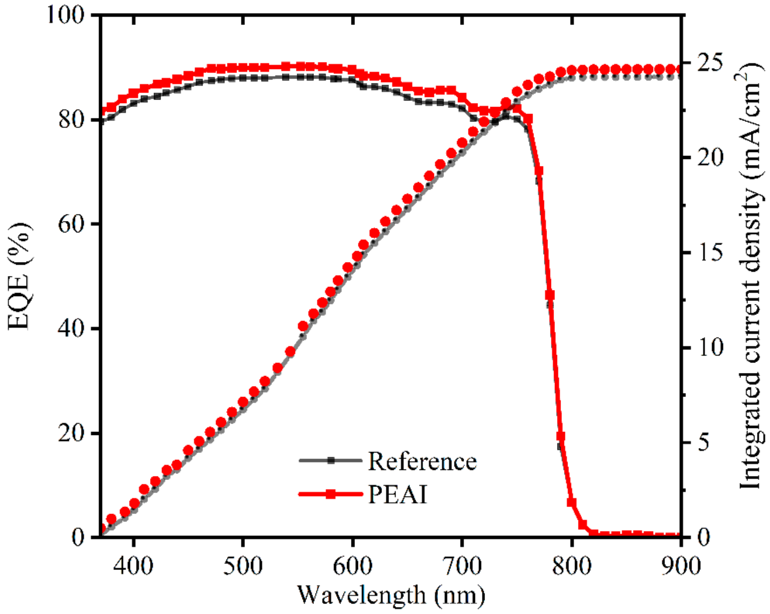

Figure S3. External quantum efficiency (EQE, left axis) and integrated current density (right axis) for reference and PEAI-passivated devices. The PEAI-treated device exhibits higher EQE across the visible spectrum, with an integrated Jsc of ~25 mA cm^{-2}, closely matching the Jsc extracted from J–V measurements.

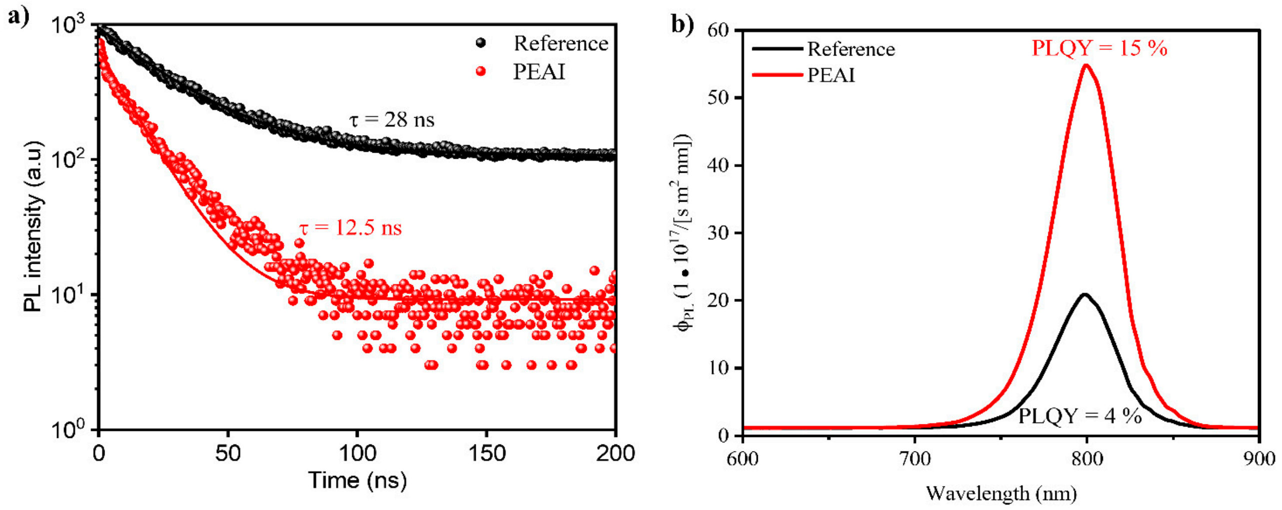

Figure S4. (a) TRPL decay showing reduced carrier lifetime with PEAI passivation. (b) PLQY increases from 4% to 15%, indicating enhanced radiative recombination and defect passivation.

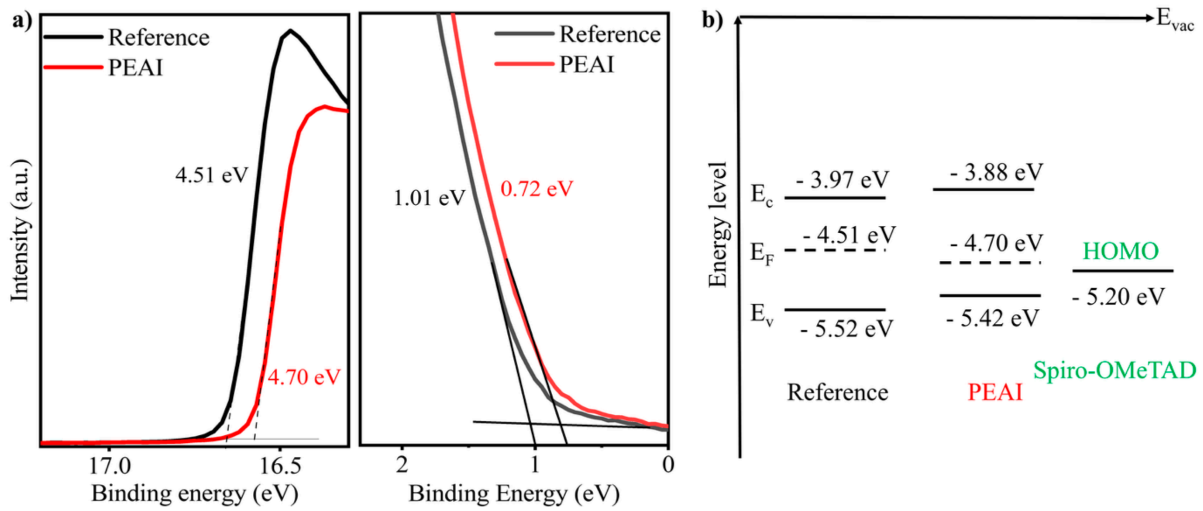

Figure S5. UPS spectra (a) and corresponding energy level diagram (b) illustrating the impact of PEAI treatment on the energy level alignment at the perovskite surface.

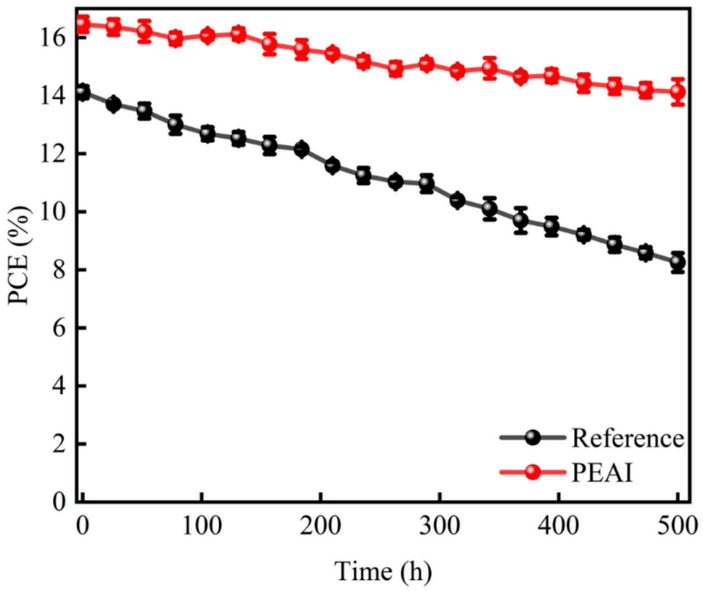

Figure S6. Thermal stability comparison between PEAI-treated and control perovskite solar cells under continuous thermal stress (500 hours) at RH ~ 25%. Efficiency values are averages measured from five independent devices per condition, with error bars representing the standard deviation. The PEAI-treated devices retain approximately 85% of their initial performance, significantly outperforming control devices, which exhibit around 40% efficiency loss.

Table S2: Performance benchmarking of the current PEAI-treated flexible perovskite solar cells compared to previously reported flexible perovskite devices with similar architectures and fabrication methods.

**Architecture**	**PCE (%)**	**Mechanical Stability (Bending cycles / Radius)**	**Environmental Resistance (Humidity / Thermal)**	**Reference**
$MAPbI_3$, two-step deposition	12.7	1000 / 5 mm	RH 50% / 60°C	Li et al.
$MAPbI_3$, hybrid vacuum-solution	14.5	1200 / 4 mm	RH 60% / 65°C	Fu et al.
$MAPbI_3$, multistage deposition	15.8	1500 / 5 mm	RH 55% / 70°C	Momblona et al.
$MAPbI_3$, vacuum-deposited p-i-n	16.5	1300 / 4 mm	RH 60% / 60°C	Feng et al.
$MAPbI_3$, vacuum-deposited n-i-p	20.0	1100 / 6 mm	RH 55% / 65°C	Wu et al.
FA-based perovskite, vacuum deposition	14.6	1600 / 3 mm	RH 65% / 70°C	Tong et al.
$FAxMA_{1-x}PbIyBr_{3-}y$, two-step deposition	21.08	1800 / 3 mm	RH 70% / 75°C	Yang et al.
$Cs_{0.05}(MA_{0.02}FA_{0.98})_{0.95}Pb(I_{0.98}Br_{0.02})_3$	20.64	1600 / 3 mm	RH 65% / 70°C	Zhao et al.
Our work	17.0	700 / 5 mm	RH 25% / 60°C	

Table S3: Comparison of PEAI passivation with recent surface treatments for flexible PSCs.

Surface Passivation	PCE (%)	Bending Radius (mm)	Bending Cycles	Efficiency Retention (%)	Reference
PMMA	17.03	4	1000	~80	Sun et al., ACS Appl. Mater. Interfaces, 2022, 14, 12003–12012.
PEABr	18.48	3	1500	~85	Zhang et al., J. Colloid Interface Sci., 2022, 608, 3151.
BAI	20.10	3	1000	~87	Wu et al., Adv. Mater., 2021, 33, 2102541.
OAI	19.50	5	1000	~83	Tan et al., ACS Appl. Mater. Interfaces, 2021, 13, 20034.
PEAI (2D/3D)	17.0	5	700	~88	**This work**

References

1. Y. Li, L. Meng, Y. Yang, G. Xu, Z. Hong, Q. Chen, J. You, G. Li, Y. Yang, Nat. Commun., 2014, **5**, 5413.
2. F. Fu, T. Feurer, T. Jäger, E. Avancini, B. Bissig, S. Yoon, S. Buecheler, A. N. Tiwari, Nat. Commun., 2015, **6**, 8932.
3. C. Momblona, L. Gil-Escrig, E. Bandiello, M. Sessolo, E. M. Hutter, K. Lederer, J. Blochwitz-Nimoth, H. J. Bolink, Energy Environ. Sci., 2016, **9**, 3456–3463.
4. J. Feng, X. Zhu, Z. Yang, X. Zhang, J. Niu, Z. Wang, S. Zuo, Adv. Mater., 2018, **30**, 1801418.
5. C. Wu, K. Wang, S. Li, S. Liang, Y. Liu, X. Wang, Z. Yu, ACS Appl. Mater. Interfaces, 2020, **12**, 13773–13780.
6. G. Tong, X. Lan, Z. Song, W. Li, H. Wang, H. Zhang, C. Chen, J. Mater. Chem. A, 2020, **8**, 5350–5360.
7. D. Yang, X. Yang, K. Wang, C. Wu, R. Zhu, X. Wang, J. Feng, Energy Environ. Sci., 2021, **14**, 3233–3255.
8. Y. Zhao, Y. Wang, X. Gao, Y. Li, L. Meng, Nat. Sustain., 2022, **5**, 515–523.
